# SPECT-OPT multimodal imaging enables accurate evaluation of radiotracers for β-cell mass assessments

**DOI:** 10.1038/srep24576

**Published:** 2016-04-15

**Authors:** Wael A. Eter, Saba Parween, Lieke Joosten, Cathelijne Frielink, Maria Eriksson, Maarten Brom, Ulf Ahlgren, Martin Gotthardt

**Affiliations:** 1 Department of Radiology and Nuclear Medicine, Radboud University Medical Center, Nijmegen, The Netherlands; 2 Umeå Centre for Molecular Medicine, Umeå University, Umeå, Sweden

## Abstract

Single Photon Emission Computed Tomography (SPECT) has become a promising experimental approach to monitor changes in β-cell mass (BCM) during diabetes progression. SPECT imaging of pancreatic islets is most commonly cross-validated by stereological analysis of histological pancreatic sections after insulin staining. Typically, stereological methods do not accurately determine the total β-cell volume, which is inconvenient when correlating total pancreatic tracer uptake with BCM. Alternative methods are therefore warranted to cross-validate β-cell imaging using radiotracers. In this study, we introduce multimodal SPECT - optical projection tomography (OPT) imaging as an accurate approach to cross-validate radionuclide-based imaging of β-cells. Uptake of a promising radiotracer for β-cell imaging by SPECT, ^111^In-exendin-3, was measured by *ex vivo-*SPECT and cross evaluated by 3D quantitative OPT imaging as well as with histology within healthy and alloxan-treated Brown Norway rat pancreata. SPECT signal was in excellent linear correlation with OPT data as compared to histology. While histological determination of islet spatial distribution was challenging, SPECT and OPT revealed similar distribution patterns of ^111^In-exendin-3 and insulin positive β-cell volumes between different pancreatic lobes, both visually and quantitatively. We propose *ex vivo* SPECT-OPT multimodal imaging as a highly accurate strategy for validating the performance of β-cell radiotracers.

The prevalence of diabetes in 2013 was 382 million and according to the 2015 International Diabetes Federation report, the number of diabetes cases has risen to 415 million^1^. Both β-cell dysfunction and death contribute to diabetes progression. The relationship between β-cell mass (BCM) and function is not clear and therefore, routine immunological and functional tests are not suitable to monitor changes in BCM^2^,^3^,^4^. There is a high interest in developing non-invasive tools to quantify BCM, independently of their function, to better understand disease progression. Current non-invasive imaging techniques, with the highest anatomic resolution available, are not powerful enough to detect single islets *in vivo* at this time. Alternatively, radionuclide-based imaging methods are used for their advanced detection sensitivity of tracer uptake by β-cells^5^. Specific targeting of β-cells by ^111^In-exendin-3 followed by Single Photon Emission Computed Tomography (SPECT) was reported as a promising tool to detect and quantify small differences in BCM in type 1 diabetes (T1D) rodent models as well as T1D patients^6^,^7^. These studies showed a linear correlation between ^111^In-exendin-3 uptake and histological quantitative analysis of BCM in rodents. Although comparison between the accumulation of the radiotracer and two-dimensional stereological techniques is a conventional research practice, which benefits from the possibility to correlate tracer uptake with the quantification of insulin producing β-cells at sub-cellular resolution, such methods are associated with several disadvantages. Typically, stereological techniques rely on certain assumptions, in particular regarding the degree of convexity of the islets and their orientation/distribution within the gland, to estimate islet β-cell volume or mass. In addition, such techniques are highly labor and time consuming and are consequently performed by intervallic or random sampling, even for limited cohorts of animals. Therefore the outcome will inevitably be an extrapolation of two-dimensional information. Islets differ in size and are heterogeneously distributed within the organ, both with respect to the individual lobes and between the lobes^8^,^9^. In addition, different regions of the pancreas could be unequally affected by diabetes in different animals of the same strain and stage^10^, further complicating the picture for histological analyses. Hence sampling techniques are, depending on the analyzed model, prone to over- or underestimate the true BCM and are largely dependent on the chosen quantification method. Indeed, the establishment of a stereological approach to describe the full pancreatic islet-volume distribution has been described as a “true stereological challenge”^11^. Consequently, an alternative strategy to correlate radiotracer uptake in the pancreas with actual BCM is needed.

Previous studies developed an Optical Projection Tomography (OPT)-based method that allows visualizing and studying fixated biological specimens at high spatial resolution^12^. This technique was remarkably accurate for quantifying BCM and determining the spatial distribution within and between the lobes of the pancreas down to the resolution of single islets after *ex vivo* antibody-based targeting of insulin^8^,^9^,^13^. Importantly, OPT allows to scan a sample with high resolution at the millimeter to centimeter-scale^14^, thereby being a technique of choice to image complete lobes of rodent pancreas^8^,^13^.

*In vivo* determination of BCM using ^111^In-exendin-3 SPECT was previously shown to correlate with *ex vivo* quantification of ^111^In-exendin-3 uptake in the same pancreas^15^. Hence, *ex vivo* SPECT acquisitions could be used as a reliable alternative to *in vivo* acquisitions to assess the performance of β-cell radiotracers. Importantly, *ex vivo* SPECT imaging offers the advantage of easy handling and positioning of the pancreas in the SPECT scanner, for accurate cross-validation of radiotracer uptake and 3D distribution with comparative *ex vivo* methods.

Against this background, we sought to determine whether fast and accurate cross-validation of radionuclide-based imaging of β-cells can be achieved by sequential *ex vivo* SPECT-OPT multimodal imaging, as compared to *ex vivo* SPECT followed by histology. Uptake of ^111^In-labeled [Lys^40^(DTPA)_6_]-exendin-3 by the β-cells was quantified by SPECT and was cross-examined by OPT-based assessment of insulin positive β-cell volumes followed by histological analysis of insulin positive β-cell areas in the splenic, gastric and duodenal lobes^14^ of healthy and alloxan-treated diabetic Brown Norway (BN) rats.

## Results

### Correlation of SPECT imaging with morphometric determination of BCM

^111^In-exendin-3 uptake was quantified by SPECT imaging of single pancreatic lobes from healthy and diabetic animals, and the accuracy of the measurements was verified by the linear correlation between SPECT data and gamma-counter based analysis of radioactivity (*r*^2^ = 0.85) (Supplementary Figure S1A). Healthy rats showed higher accumulation of radioactivity (measured by gamma-counter) per gram of pancreatic tissue when compared to the diabetic group (Supplementary Figure S2A).

**Figure 1:**
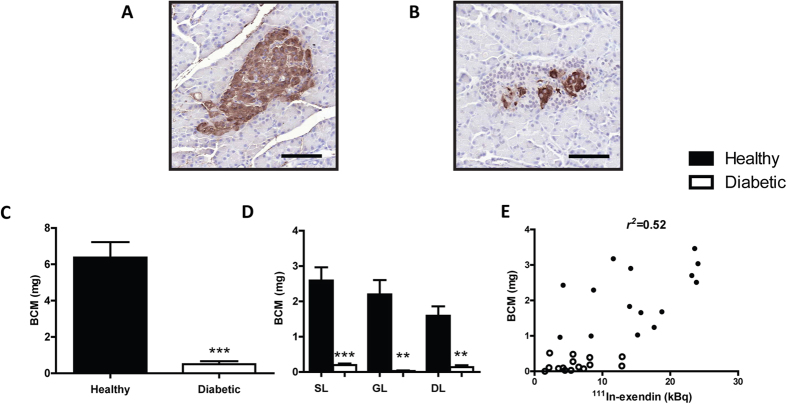
Histological quantitative analysis of BCM correlates with ^111^In-exendin-3 uptake. (**A**,**B**) Representative images of insulin positive areas in control (**A**) and alloxan treated (**B**) rats. (**C**) Bar chart displaying BCM (mg) (n = 5). (**D**) Graph displaying quantitative analysis of β-cell inter-lobular distribution (n = 4–5). (**E**) BCM (mg) was plotted against ^111^In-exendin-3 uptake (kBq, kilobecquerels) as determined by SPECT (*r*^2^ = 0.52, p = 8.13 × 10^−06^). In (**A**–**E**) untreated rats are shown in black and alloxan-treated rats are shown in white. Data are shown as means ± SEM, where *p < 0.05, **p *<* 0.01 and ***p *<* 0.001 compared with the corresponding healthy group. Scale bar represents 100 μm.

Morphometric analysis of insulin staining was conducted at three different levels of each pancreatic lobe (Fig. 1A,B). Insulin stained BCM (mg) in alloxan treated pancreata was significantly reduced to 7.94% in comparison with the healthy group (p < 0.001) (Fig. 1C). Spatial quantitative analysis of β-cells did not show any significant differences in their distribution between the pancreatic lobes (Fig. 1D). In line with our previous studies^7^, we observed a linear correlation between ^111^In-exendin-3 uptake and insulin positive area per lobe (*r*^2^ = 0.52) (Fig. 1E). Similarly, a linear correlation was observed between SPECT and histological based analysis of total BCM (*r*^2^ = 0.53) (Supplementary Figure S1B).

### Multimodal imaging of rat pancreatic lobes with SPECT and OPT

**Figure 2:**
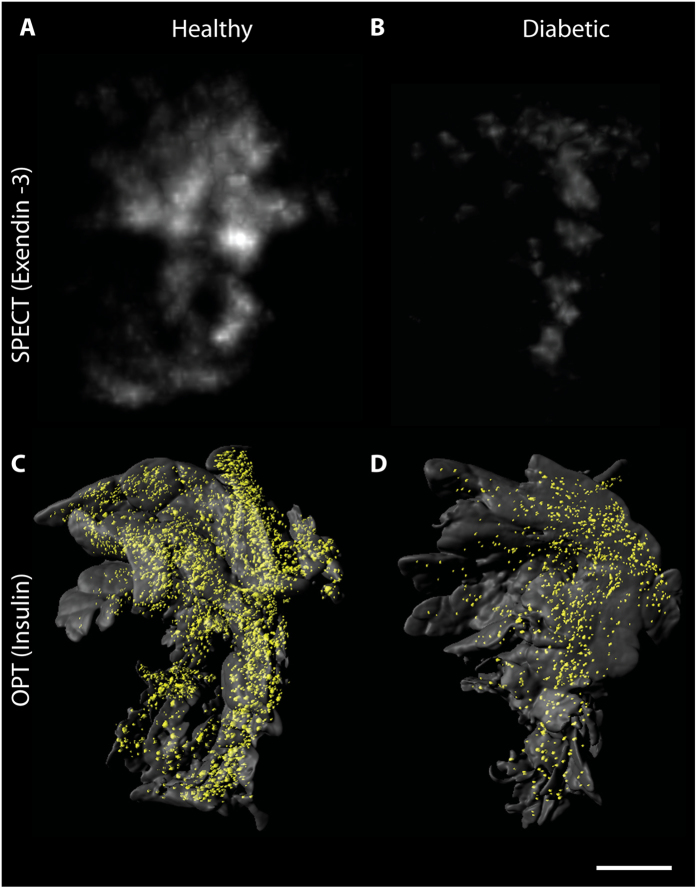
Multimodal imaging of pancreatic β-cells with SPECT and OPT. (**A**,**B**) *Ex vivo* SPECT scans of representative splenic lobes from a healthy (**A**) and an alloxan treated animal (**B**) respectively. (**C**,**D**) OPT generated iso-surface images of the same lobes as visualized in (**A**,**B**). Alloxan-treated rats exhibit lower ^111^In-exendin-3 uptake and β-cell volume when compared to the control group. Scale bar represents 3 mm.

**Figure 3:**
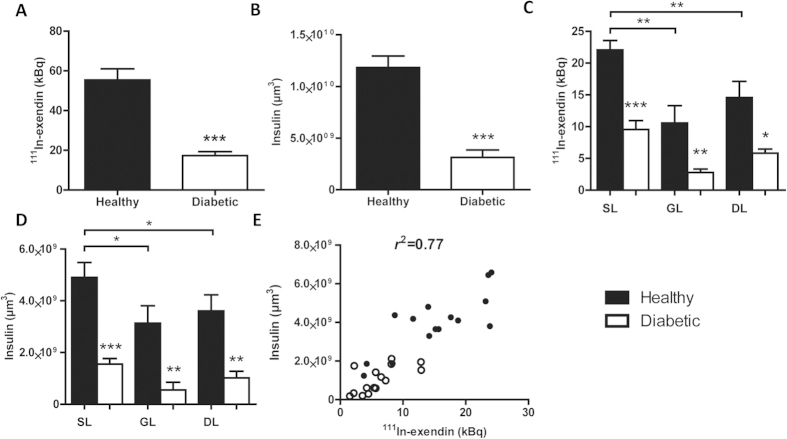
SPECT based radioactive quantification of ^111^In-exendin-3 uptake correlates with OPT based quantification of insulin positive β-cell volume. (**A**,**B**) Quantification of absolute ^111^In-exendin-3 uptake (kBq) by SPECT (**A**) and total β-cell volume (μm^3^) by OPT (**B**) (n = 5). (**C**,**D**) Graphs illustrating the heterogeneous distribution of β-cells between the splenic, gastric (GL) and duodenal lobes (DL) as determined by SPECT (**C**) and OPT (**D**) respectively (n = 4–5). (**E**) Graph showing pancreatic uptake of ^111^In-exendin-3 by separate lobes plotted against β-cell volume shows a strong correlation (*r*^2^ = 0.77, p = 2.07 × 10^−10^). In (**A**–**E**) untreated rats are shown in black and alloxan-treated rats are shown in white. Data are shown as means ± SEM, where *p < 0.05, **p *<* 0.01 and ***p *<* 0.001 compared with the corresponding healthy group.

High uptake of ^111^In-exendin-3 was observed by SPECT in the pancreas of healthy rats (Fig. 2A). This was in line with the abundant insulin staining visualized by OPT (Fig. 2C). Rats that were treated with alloxan prior to SPECT and OPT imaging exhibited decreased signal by both modalities (Fig. 2B,D). Furthermore, the distribution of ^111^In-exendin-3 accumulation was similar to the distribution of the β-cells observed by OPT. Quantification of ^111^In-exendin-3 uptake showed an accumulation of 55.46 ± 5.55 kBq in the pancreas and a total β-cell volume of 1.18 × 10^10^ ± 1.11 × 10^9^ μm^3^ (Fig. 3A,B). Quantitative analysis of alloxan-treated rats showed a significant decrease in BCM (p < 0.001), where uptake of ^111^In-exendin-3 (17.27 ± 2.04 kBq) and β-cell volume (3.13 × 10^9^ ± 7.17 × 10^8^ μm^3^) dropped to 23% and 26.5%, respectively, when compared to the healthy group. Quantification of ^111^In-exendin-3 uptake in splenic, gastric and duodenal lobes, showed inhomogeneous distribution of ^111^In-exendin-3 accumulation between the lobes, as the uptake was 22.06 ± 1.48, 10.58 ± 2.72 and 14.57 ± 2.55 kBq, respectively (Fig. 3C). A similar distribution pattern was observed by OPT, where β-cell volume in splenic, gastric and duodenal lobes was equal to 4.90 × 10^9^ ± 5.81 × 10^8^, 3.14 × 10^9^ ± 6.72 × 10^8^ and 3.61 × 10^9^ ± 6.25 × 10^8^ μm^3^, respectively (Fig. 3D). Alloxan treatment resulted in decreased ^111^In-exendin-3 uptake (measured by SPECT) and β-cell volumes (measured by OPT) in all lobes. The linear correlation (*r*^2^) between ^111^In-exendin-3 uptake and β-cell volume per lobe was 0.77 (Fig. 3E), and for total BCM 0.81 (Supplementary Figure S1C).

Importantly, SPECT data was successfully compared with OPT analysis of the entire islet population of each pancreas within a time period during which only a limited number of intervallic sections per lobe could be analyzed by stereology. Furthermore, SPECT-OPT correlation (*r*^2^ = 0.77) was significantly superior to SPECT-histology (*r*^2^ = 0.52) (p < 0.001). There were no significant differences between the linear regression slopes (p = 0.82) (Supplementary Figure S2B).

## Discussion

In the present study we examined the utility of SPECT-OPT multimodal imaging for fast and accurate validation of radionuclide-based imaging of β-cells. The successful imaging of β-cells by SPECT and OPT demonstrated the possibility of combining the protocols of both imaging modalities. ^111^In-exendin-3 uptake and insulin positive β-cell volume as measured by SPECT and OPT respectively, were in excellent linear correlation. These results demonstrate that the performance of β-cell radiotracers can be cross-examined by OPT.

While the resolution provided by conventional microscopy prevails over OPT, correlation of insulin staining with ^111^In-exendin-3 uptake by immunohistochemical analysis was significantly lower, indicating that stereological methods may be less accurate than OPT for validating SPECT-based quantification of BCM, mainly because they are prone to BCM quantification errors. Including more histological sections into the quantitative analysis for each lobe might increase the BCM quantification accuracy, but would also be more labor and time-consuming. In contrast, an excellent linear correlation was achieved in a similar period of time, by combining SPECT and OPT imaging, where quantification of tracer uptake was followed by a single acquisition of the entire insulin positive β-cell population of intact pancreatic lobes^8^, instead of extrapolating from 2D data.

Histological assessment of islet spatial heterogeneity is challenging, given the 2D aspect of the method. On the other hand, differences in distribution of the islets between the pancreatic lobes were evident by SPECT and OPT. The splenic lobe harbored the largest BCM, followed by the duodenal and the gastric lobes respectively, which is in agreement with the β-cell distribution previously recorded for the murine pancreas^9^. Furthermore, the distribution of ^111^In-exendin-3 in alloxan treated pancreata was quantitatively, and visually in agreement with OPT (Fig. 2). Residual β-cells in alloxan-treated rats were mainly observable in the core of the lobes, an area also showing the highest accumulation of ^111^In-exendin-3. The high correspondence between SPECT and OPT information confirms the previously reported radiotracer specificity towards the β-cells for ^111^In-exendin-3^7^, indicating that SPECT-OPT is a highly accurate and convenient alternative to SPECT-histology when characterizing β-cell radiotracers for BCM assessments. Hence, the correlation value between SPECT and OPT could reflect the performance of the radiotracer under investigation.

The possibility to visualize the individual lobes by *in vivo* SPECT has not been demonstrated, and with current technologies must be seen as a challenging undertaking. Visualizing of the complete pancreas of rodents is highly challenging with any *in vivo* imaging modality available, let alone resolving the individual lobes. However, as previously been demonstrated there is a very good correlation of total pancreatic BCM between *in vivo* and *ex vivo* analysis^15^. In the present study, *ex vivo* SPECT-OPT multimodal imaging allowed validation of radiotracer biodistribution in the pancreas and included, for the first time, information with respect to β-cell spatial heterogeneity, which renders this methodology highly convenient when validating established radiotracers and evaluating the specificity and performance of novel β-cell radiotracers.

In conclusion, we report the successful multimodal imaging of pancreatic islets after coupling protocols from two independent approaches into one that combines radionuclide and optical imaging of β-cells. The higher correlation between ^111^In-exendin-3 uptake and β-cell volume obtained by SPECT-OPT, compared to the correlation between ^111^In-exendin-3 uptake and β-cell area by SPECT-histology, indicates that this new strategy may be more reliable in validating the actual performance of β-cell radiotracers for BCM assessments.

## Methods

### Animals and alloxan treatment

Female Brown Norway rats (110–120 g) were purchased from Charles River (Calco, Itlay). All experiments were conducted in accordance with Radboud Univserity and Umeå University guidelines and on humane care and use of laboratory animals. Experiments were approved by the Animal Ethical Committee of the Radboud University, Nijmegen, The Netherlands and the Ethical Committee for Animal Research, Northern Sweden. Rats assigned to the diabetic group were injected intravenously with 60 mg/kg of alloxan (Sigma, St Louis, MO, USA) as previously described^7^ and animals assigned to the healthy group were injected with the vehicle compound. Experiments started after confirmation of hyperglycemia (>20 mmol/L) in alloxan-treated rats by using a glucose meter (Accu-Chek Sensor; Roche Diagnostics; Almere, The Netherlands).

### Radiolabeling of exendin-3

[Lys^40^(DTPA)_6_]-exendin-3 was purchased from Peptide Specialty Laboratories (Heidelberg, Germany). Peptide labeling with In-111 and quality controls were performed as previously described^7^. All animals were injected with 20 pmol of ^111^In-exendin-3 corresponding to approximately 150 MBq.

### SPECT imaging and data acquisition

Rats were intravenously injected with radio-labeled ^111^In-exendin-3 and euthanized 1 hour post-injection. The pancreata were isolated and the splenic, gastric and duodenal lobes were separated. Finally, lobes were fixed in 4% PFA (w/v in phosphate buffered saline (PBS)) for two hours and were scanned in PBS with the small animal U-SPECT-II/CT system (MILabs, Utrecth, The Netherlands) with a 0.2 mm multi-pinhole collimator for 12 hours. Images were reconstructed with OSEM (3 iterations, 16 subsets, voxel size 2 mm) using the U-SPECT-Rec software (MILabs, Utrecht, The Netherlands) and the measured counts were converted to kBq (Kilobecquerels) using standards with known radioactivity concentrations scanned with the same settings. After SPECT acquisitions, lobes were measured for radioactivity in a γ-counter (Wallac 1480 Wizard, Perkin Elmer, Boston, MA, USA) and data were expressed as the percentage of administered dose.

### OPT imaging and data acquisition

After quantification of radioactivity, pancreata were stepwise dehydrated in 33%, 66% and 100% methanol (v/v in PBS) and stored at −20 °C. Tissue samples were next stained for Insulin (Primary antibody Guinea Pig anti-insulin, (DAKO: A0564) and secondary antibody IRDye 680 anti-Guinea Pig (Licor: 926–68077)) and processed for OPT imaging essentially as described previously^13^. OPT scans were performed for individual lobes using a Near Infrared-OPT (NIR-OPT) setup with excitation filter 665/45 nm and emission filter 725/50 nm as described previously^13^. The anatomy of the pancreas, based on its autofluorescence, was obtained by scanning it with a excitation 480/40 nm and emission 510LP filter set. Tomographic reconstruction was performed essentially as described^13^. Contrast limited adaptive histogram equalization (CLAHE) was not required due to a good signal to noise ratio obtained in volume rendering data. Insulin stained β-cell volume was quantified by generating 3D iso-surface based on signals from individual islets using Imaris 7.7. software (Bitplane).

### Immunohistochemistry and morphometric analysis

Following OPT scans, the specimens were treated with 100% methanol 4–5 times (for 3–4 days) and were rehydrated with 70%, 50%, 30% and 10% ethanol (v/v in MQ). Excess agarose was removed when the samples were in 10% ethanol and then incubated in 0.29 M sucrose for 60 min at room temperature followed by incubation in preheated 0.29 M sucrose at 57 °C to completely remove residual agarose and washed in PBS. Next, the pancreata were embedded in paraffin and 4 μm thick sections were cut at 3 separate levels of the tissue with 100 μm distance. Sections were processed for antigen retrieval by incubating in 10 mM citrate pH 6 for 10 min at 96 °C and blocked with 1% BSA (w/v in PBS) for 30 min and endogenous peroxidase activity was blocked with 3% H_2_O_2_ (v/v in PBS) for 30 min. Staining was performed using anti-insulin rabbit antibody (Santa Cruz Biotechnology, Santa Cruz, CA, USA) diluted 1/50 for 1 hour at room temperature and washed in PBS. Sections were next incubated with swine anti-rabbit conjugated with peroxidase, for 30 min at room temperature and washed with PBS. Finally, hematoxylin counterstaining was performed and sections were scanned with Pannoramic250 Flash II scanner (Budapest, Hungary). To quantify BCM (mg), β-cell area in pancreatic sections was estimated by drawing regions of interest around insulin-positive areas and was divided by the total pancreatic section (insulin positive fraction). BCM estimation was finally obtained by multiplying the insulin positive fraction by the pancreatic lobe weight. Area quantification was performed using Adobe Photoshop CS5 Version 12.0.

### Statistical analysis

Number of animals was five for each condition. Values are expressed as means ± SEM. Student t-test was performed for comparisons, where P value < 0.05 was considered as statistically significant. To assess whether the correlations of SPECT-OPT and SPECT-histology differ significantly, comparisons were performed using two-way dependent correlation coefficients comparison. Linear regression slopes were compared using multiple linear regression analysis (ANCOVA). Statistical tests were performed with Graphpad Prism 5 (GraphPad Software version 5.03, San Diego California USA) or SPSS software (Version 22.0. Armonk, NY, IBM Corp).

## Additional Information

**How to cite this article**: Eter, W. A. *et al.* SPECT-OPT multimodal imaging enables accurate evaluation of radiotracers for β-cell mass assessments. *Sci. Rep.* **6**, 24576; doi: 10.1038/srep24576 (2016).

## Supplementary Material

Supplementary Information
