# Supplementary material for: SPECT-OPT multimodal imaging enables accurate evaluation of radiotracers for β-cell mass assessments
Source: Sci Rep. 2016 Apr 15;6:24576. doi: 10.1038/srep24576 (PMC4832194; doi:10.1038/srep24576)
Supplement: Supplementary Information [file srep24576-s1.pdf]

Title : **SPECT-OPT multimodal imaging enables accurate evaluation of radiotracers for β-cell mass assessments**

Authors: Wael A Eter[1†*], Saba Parween[2†], Lieke Joosten[1], Cathelijne Frielink[1], Maria Eriksson[2], Maarten Brom[1], Ulf Ahlgren[2#], Martin Gotthardt[1#]

[1]Department of Radiology and Nuclear Medicine, Radboud University Medical Center, Nijmegen, The Netherlands
[2]Umeå Centre for Molecular Medicine, Umeå University, Umeå, Sweden
[†]These authors contributed equally to this study
# Co-senior authorship
* Corresponding author

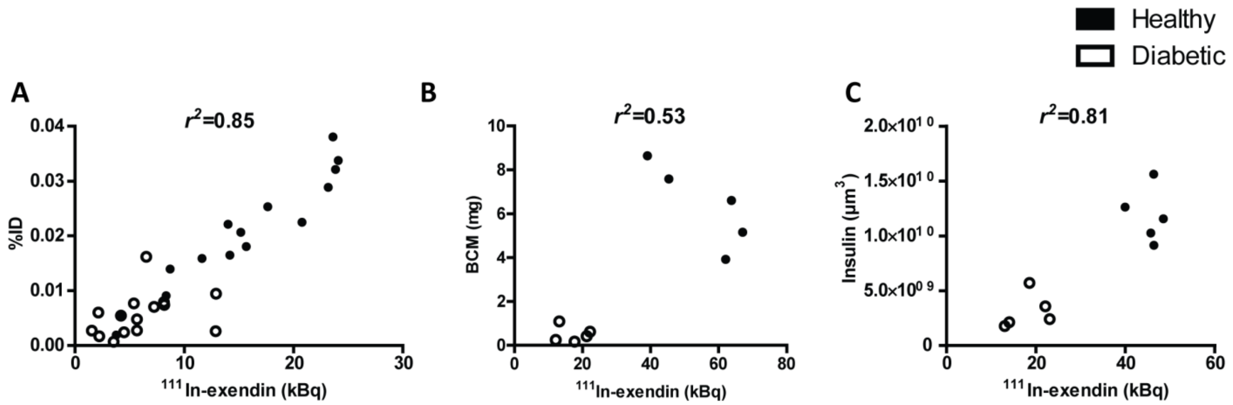

Figure S1. Linear correlations between ^{111}In-exendin-3 uptake and BCM. A, Correlation of SPECT data (kBq) with gamma-counter analysis (%ID, percentage of injected dose) of separate pancreatic lobes (r^2=0.85, p=5.55x10^{-12}) (n=15)**. B and C,** graphs showing total pancreatic uptake of ^{111}In-exendin-3 (SPECT) plotted against total BCM (histology) (r^2=0.53, p=0.017) and total pancreatic β-cell volume (OPT) (r^2=0.81, p=0.0004) respectively (n=5).

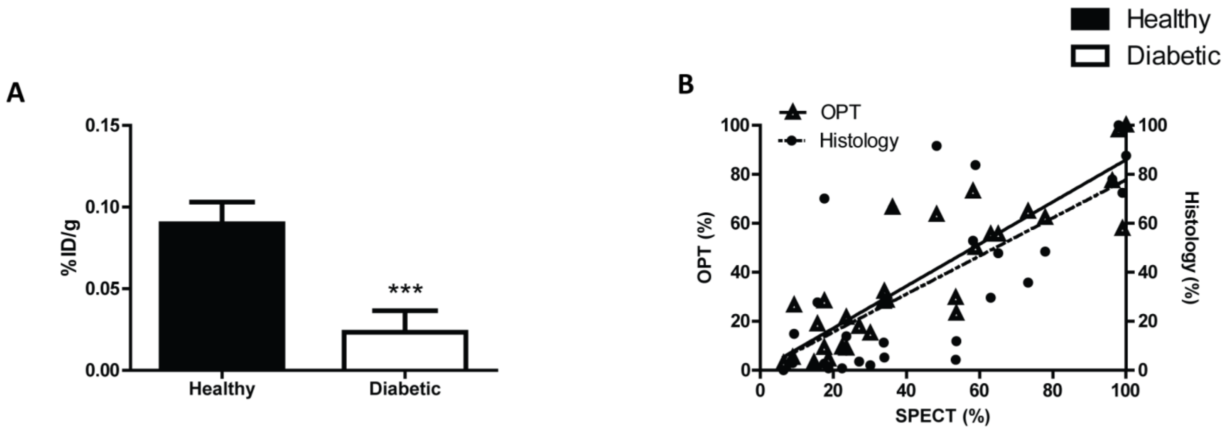

Figure S2. Alloxan treated rats display a significantly reduced uptake of ^{111}In-exendin-3. A, The Graph illustrates the pancreatic uptake of radioactivity normalized by the weight of the pancreas (%ID/g, percentage of injected dose per gram) of healthy control animals and alloxan treated animals as measured by gamma-counter. **B**, Two-way dependent correlation coefficients comparison (SPECT-OPT r^2=0.77, SPECT-histology r^2=0.52; n=30) reveals significant differences between SPECT-OPT and SPECT-histology correlations ($p<0.001$). ANCOVA analysis reveals no significant differences between SPECT-OPT and SPECT-histology linear regression slopes (p=0.82). Data are expressed in relation to (%) the highest BCM value measured in a lobe by the corresponding modality.
